# Utilizing Massively Parallel Sequencing (MPS) of Human Leukocyte Antigen (HLA) Gene Polymorphism to Assess Relatedness in Deficiency Parentage Testing

**DOI:** 10.3390/genes15020150

**Published:** 2024-01-24

**Authors:** Diamanto I. Kouniaki, Konstantinos V. Fotopoulos, Katerina Tarassi, Alexandra Tsirogianni

**Affiliations:** 1 Immunology and Histocompatibility Department, Evangelismos General Hospital, 10676 Athens, Greece; kouniakitzeni@yahoo.gr (D.I.K.); katerinatarassi@gmail.com (K.T.); 2 School of Electrical and Computer Engineering, National Technical University of Athens (ECE-NTUA), 15772 Zografou, Greece; kostfoto2001@gmail.com

**Keywords:** forensic medicine, aSTR mutation, parentage testing, HLA alleles, massively parallel sequencing technology

## Abstract

In the realm of DNA testing with legal implications, the reliability and precision of genetic markers play a pivotal role in confirming or negating paternity claims. This study aimed to assess the potential utility of human leukocyte antigen (HLA) gene polymorphism through massively parallel sequencing (MPS) technology as robust forensic markers for parentage testing involving genetic deficiencies. It sought to redefine the significance of HLA genes in this context. Data on autosomal short tandem repeat (aSTR) mutational events across 18 paternity cases involving 16 commonly employed microsatellite loci were presented. In instances where traditional aSTR analysis failed to establish statistical certainty, kinship determination was pursued via HLA genotyping, encompassing the amplification of 17 linked HLA loci. Within the framework of this investigation, phase-resolved genotypes for HLA genes were meticulously generated, resulting in the definition of 34 inherited HLA haplotypes. An impressive total of 274 unique HLA alleles, which were classified at either the field 3 or 4 level, were identified, including the discovery of four novel HLA alleles. Likelihood ratio (LR) values, which indicated the likelihood of the observed data under a true biological relationship versus no relationship, were subsequently calculated. The analysis of the LR values demonstrated that the HLA genes significantly enhanced kinship determination compared with the aSTR analysis. Combining LR values from aSTR markers and HLA loci yielded conclusive outcomes in duo paternity cases, showcasing the potential of HLA genes and MPS technology for deeper insights and diversity in genetic testing. Comprehensive reference databases and high-resolution HLA typing across diverse populations are essential. Reintegrating HLA alleles into forensic identification complements existing markers, creating a potent method for future forensic analysis.

## 1. Introduction

Human leukocyte antigens (HLAs) are part of the major histocompatibility complex (MHC) and play an important role in the regulation of the immune system, as well as in fundamental molecular and cellular processes [1]. The HLA system is one of the most polymorphic regions of the human genome and, to date, more than 36,000 HLA class I and II alleles have been identified according to the Immuno Polymorphism Database-ImMunoGeneTics project/HLA Database (IPD-IMGT/HLA database, v 3.54, 2023-03), which contains coding for more than 21,500 distinct functional proteins specialized in presenting antigenic peptides to the T-cell receptor (TCR) [2]. Due to their proximity on the short arm of chromosome 6 (6p21.3), a complete set of alleles of genes mapped in a row to the same chromosome is usually inherited as a haplotype in a Mendelian fashion from each parent [3]. During the 1980s to the 1990s, the determination of the HLA region served as the established procedure in forensic genetics [4]. The HLA system was considered highly suitable for parentage determination due to its low recombination rate, lack of mutation recording in family studies, and availability of HLA allele frequencies for diverse ethnic groups [5]. However, traditional HLA typing methods, which focus on the polymorphic regions responsible for encoding the antigen recognition site (ARS), namely, exons 2 and 3 for HLA-class I genes and exon 2 for HLA-class II genes, often provide ambiguous results, particularly in instances of heterozygosity. Multiple analysis patterns within the same exon coding for the HLA antigen were observed in such cases. The limited discriminatory power, which is influenced by linkage disequilibrium and prevalent alleles in certain ethnic groups, posed challenges in forensic genetics. As a result, advancements in autosomal short tandem repeat (aSTR) typing technology have largely overshadowed HLA typing in the past two decades, offering improved accuracy and discriminatory power [6,7,8,9,10].

In contemporary parentage investigations, microsatellites have emerged as the markers of choice due to their highly polymorphic nature, codominant inheritance, and wide distribution throughout the euchromatic genome [11]. Microsatellites, or autosomal short tandem repeats (aSTRs), consist of tandem repeats of short nucleotide sequences (1–6 bp repeat units) that form series with lengths of up to 100 nucleotides (nt) [12]. The Mendelian segregation of aSTR markers in families renders them ideal for personal identification systems in medical and forensic applications, where they are admissible as evidence in legal proceedings. However, certain factors can render these markers suboptimal for parentage analyses in some cases. In particular, the presence of undetectable alleles (null or silent) [13], genotyping errors, and mutational events [12,14] can lead to Mendelian-inconsistent genotypes (observed in equal different parent mismatches) between parents and offspring, potentially impacting the accuracy of genetic profiles. A null or silent aSTR allele refers to an allele at a specific genetic locus that does not produce a detectable product during the genotyping process. It may be a result of mutations or variations in the DNA sequence that impede the amplification or detection of the targeted region [13]. Genotyping errors encompass a range of inaccuracies that can occur during the process of determining an individual’s genetic composition. These errors may result from technical issues, such as equipment malfunctions, contamination, or human errors in sample handling [12,14]. Mutational events involve changes in the DNA sequence of a specific locus over time. These changes can include insertions, deletions, or substitutions of nucleotides [12,14]. Mutational events, which are primarily driven by DNA polymerase slippage, can cause variations in the microsatellite length [12,15,16]. Although the repeat length is typically maintained within a stable range during somatic or germinal replication through mismatch repair systems, sporadic gametic mutations can occur, thereby influencing the interpretation of tests. It is well established that certain alleles within a single STR locus are more prone to mutation than others in the human genome, with their genetic diversity ranging from around 10^−6^ to 10^−2^ nt per generation [12,17]. Therefore, understanding the mutation rate of locus-specific aSTRs is crucial in paternity or kinship assessments. In the context of genetic analysis, microsatellites may be considered insufficient in certain cases due to their limitations, prompting the use of alternative markers, such as hypervariable regions of mitochondrial DNA (mt-DNA) [18], single nucleotide polymorphisms (SNPs) [19], X/Y-STR analysis [20], or HLA genotyping [7], to enhance the precision and comprehensiveness of genetic profiling. Additionally, statistical approaches, which are exemplified by the formulas proposed by the AABB, offer another valuable avenue for resolving paternity tests. This comprehensive approach aims to minimize errors and ensure accurate parentage evaluation, providing a broader scope for forensic genetic analysis.

Massively parallel sequencing (MPS) technology or next-generation sequencing (NGS) technology is a transformative method in genomics that is revolutionizing HLA genotyping and enabling profound insights into genetic diversity. MPS allows for the in-depth analysis of HLA sequences, overcoming traditional method limitations [21,22]. It employs long-range PCR and phase-defined sequencing, generating extensive data for exploring unsequenced HLA regions, identifying novel alleles, and reducing per-sample costs through multiplexing [23]. This technology has widespread applications, contributing significantly to groundbreaking discoveries in various fields [24].

The present study provided information on mutational events identified in 16 aSTRs loci commonly employed in forensic and paternity testing. Particularly, within the dataset of 428 disputed parentage tests involving 1210 individuals, we observed a total of eighteen aSTR mutational events. These mutations were distributed across various loci, with five occurring on the SE33 locus; three on the vWA locus; three on the D12S391 locus; two on the D8S1179 locus; and one each on the D10S1248, D3S1358, FGA, D2S1338, and D19S433 loci. These mutational events were identified in an equivalent number of distinct parent–offspring allelic transfers, and notably, two cases involved motherless scenarios. These mutational events resulted in discrepancies in the inherited alleles between the parents and offspring, contributing to diminished residual paternity indexes (PIs) in instances where alleles at certain aSTR genetic loci are infrequent. To enhance the result reliability, an alternative approach was adopted to investigate paternity determination through HLA genotyping using MPS technology. This adjustment aimed to address potential challenges and explore additional avenues for a more comprehensive analysis. The principal objective of this research endeavor was to assess the potential of HLA gene polymorphism through the application of MPS technology, with a view toward establishing HLA genes as effective forensic markers in the realm of genetic deficiency parentage testing. This study sought to redefine the role of HLA genes within this specialized domain.

## 2. Materials and Methods

### 2.1. Sample Collection and DNA Extraction

Among 428 civil disputed parentage tests (1210 individuals) addressed to the Immunology and Histocompatibility Lab of Evangelismos General Hospital between January 2014 and May 2023, 18 aSTR mutation events were observed in an equal number of investigations without ruling out fatherhood. In all cases requested by private or court order and apart from the child in question, both parents were submitted for testing, except 74 of which were father–motherless ones (duo cases), to prove or disprove suspected hypotheses. Genomic DNA (gDNA) was isolated from peripheral blood samples collected in EDTA-containing tubes and/or oral cotton buccal swabs from all individuals using the Maxwell^®^ 16 Blood DNA Purification kit and Maxwell^®^ 16 Buccal Swab LEV DNA kit, respectively (Maxwell Promega, Madison, WI, USA). The extracted DNA was quantified and the purity (A260/280 ratio > 1.8) was confirmed using the Qubit 1X dsDNA High-Sensitivity Assay Kit (Thermo Fisher Scientific, San Francisco, CA, USA) before genotyping according to the manufacturer’s instructions.

The study was approved by the Institutional Review Board (Ethics Committee) of Evangelismos Hospital (protocol code 78 and date of approval 10 March 2023). All participants provided written informed consent for the genetic studies prior to the sample collection, in accordance with the Code of Ethics of the World Medical Association (Declaration of Helsinki). Written informed consent was also provided by a parent or legal guardian for the minor child in question.

### 2.2. CE-Based aSTR Genotyping

A total of 1210 individuals (354 trio/74 duo parentage cases) were typed in the genetic marker system composed of the 16 aSTR loci (D10S1248, vWA, D16S539, D2S1338, D8S1179, D21S11, D18S51, D22S1045, D19S433, TH01, FGA, D2S441, D3S1358, D1S1656, D12S391, SE33) plus amelogenin for gender identification, applying the AmpFlSTR^®^ NGM SElect™ PCR amplification kit (Applied Biosystems, Waltham, MA, USA) according to the manufacturer’s instructions. Amplified aSTR fragments were separated via capillary electrophoresis (CE) on an ABI 3130 Genetic Analyzer (Applied Biosystems, Waltham, MA, USA). The raw sequencing data (.fsa file) were stored using Data Collection Software v.1.3 (Applied Biosystem, Waltham, MA, USA). Microsatellite fragment analysis and allele calling were automatically assigned using GeneMapper ID-X Software v.1.3 (Applied Biosystem, Waltham, MA, USA). To avoid possible influence caused by genotyping errors, all loci with suspicious alleles were re-genotyped. The aSTR genotyping was performed according to the revised guidelines from the scientific group on DNA analysis methods (SWGDAM) and the International Society for Forensic Genetics (ISFG) recommendations [25] concerning STR nomenclature and working practices.

### 2.3. NGS-Based HLA Genotyping

#### 2.3.1. HLA Gene Amplification and Library Preparation

**Figure 1:**
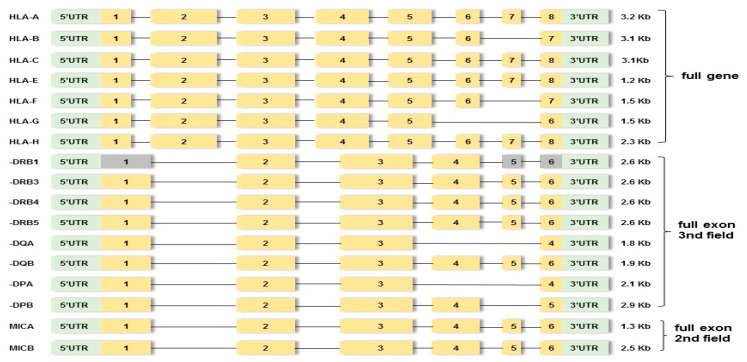
Outline of targeted PCR regions in seventeen HLA loci. Yellow boxes indicate amplified exons. Gray boxes indicate non-amplified exons. Green boxes indicate upstream UTR or 5′ untranslated region and downstream UTR or 3′ untranslated region. The region between exons involves introns.

In order to check the paternity affiliation in 18 cases with observed mutation events, further analysis of 52 individuals via amplifying 17 linked HLA loci (classical *HLA-A*, *-B*, *-C*, *-DRB1/3/4/5*, *-DQA1*, *-DQB1*, *-DPA1*, and *-DPB1*; non-classical *HLA-F*, *-G*, *-H*, and *-E* genes; and *MICA* and *MICB*) at high-resolution (HR) level using MPS assay was performed. Particularly, library preparation was carried out using hybrid capture technology supplied by an AlloSeq Tx17 kit (CareDX, Stockholm, Sweden). The multiplex commercial locus-specific primers were designed to amplify the HLA class Ia (*HLA-A*, *-B*, *-C*) and HLA class Ib (*HLA-F*, *-G*, *-H*, *-E*) from the 5′ untranslated region (UTR) to the 3′UTR (full gene). The HLA class II genes (*DRB1/3/4/5*, *-DQA1*, *-DQB1*, *-DPA1*, and *-DPB1*) were sequenced from exon 1 to the 3′UTR region (full exon to the 3rd field) and the MICA and MICB genes full exon to the 2nd field (Figure 1). Libraries were quantified using the Qubit 1X dsDNA High-Sensitivity Assay Kit (Thermo Fisher Scientific, San Francisco, CA, USA). All steps, including target generation, library preparation, clonal amplification, sequencing, and data analysis, were performed according to the manufacturer’s recommendations (Illumina, San Diego, CA, USA).

#### 2.3.2. HLA Sequencing

Sequencing was performed on an Illumina MiSeq platform (Illumina, San Diego, CA, USA). The obtained raw sequencing data (FASTQ files) were analyzed using AlloSeq Assign analysis software Tx17.1 v.1.0.4. (CareDX, Stockholm, Sweden) with reference to the IPD-IMGT/HLA database v3.51.0.0 (12 January 2023). The HLA genotyping was carried out by observing the quality metrics for each locus, including the depth of reading coverage threshold, the level of overlap to determine the phase, the coverage level of key exons, and the flag messages highlighted by the software. Alleles were described with the first 3 fields of HLA allele nomenclature, which represent the nucleotide level assignment, or the 4th field (full gene).

When a potentially novel allele was detected, confirmatory HLA typing was performed using a multiplex long-range PCR assay using a commercial NGSgo^®^-MX6-1 kit (GenDx, Utrecht, The Netherlands), and the sequencing was carried out on a MiSeq system (Illumina, San Diego, CA, USA). The obtained sequencing data were analyzed using NGSengine software v.2.29.0 (GenDx, Utrecht, The Netherlands) with reference to the IPD-IMGT/HLA Database v3.51.0.0 (12 January 2023).

### 2.4. Statistical Analysis

#### 2.4.1. Data Analysis for aSTRs Markers

In this study, in order to establish the kinship relationship between the parents and the child in question using aSTRs, the statistical parameters of power of exclusion (PE), random man not excluded (RMNE) [26], paternity index (PI) value of each locus, cumulative paternity index (CPI) value for all loci, and the probability of paternity (W) were performed by applying Bayes’ theorem [27] and according to the guidelines and recommendations of the International Society for Forensic Genetics (ISFG) [25]. Additionally, in order to create pedigrees and calculate the CPI and W values, the “Familias” program (downloaded free from http://www.nr.no/familias, accessed on 1 May 2010) was used. The likelihood ratio calculations (LRs) were based upon aSTR allelic frequencies, as estimated from the Caucasian population database provided by Steffen C.R. et al. [28]. Then, the likelihood ratio (LR) values, which represent the ratio of the likelihood of the observed data under the hypothesis of a true biological relationship to the likelihood under the hypothesis of no relationship, were subsequently calculated.

According to the American Association of Blood Banks (AABB) guidelines, in order to avoid the risk of falling into a false exclusion of the biological father of the child, more than two mismatches are required to satisfy the principle for an unambiguous exclusion of paternity [29]. In cases where 1 or 2 isolated exclusions occur for PI computation, the AABB recommends employing the corresponding mutation rate (μ) and the average probability of exclusion (PE) for non-fathers within the given system, as expressed in the formula PI = μ/PE [25]. The mutation rate (μ) was estimated using the observed frequency of inferred mutations at that marker in casework triplets, as expressed in the formula μ = s/n, where n is the total number of meiosis events and s is the number of these events deemed to be mutations [30]. An estimation of the germ-line mutation at genetic loci can be achieved by comparing the genotypes of offspring to those of their parents, after discarding genotyping errors, and is typically recognized as a shift in allelic mobility. The combined PI was calculated by multiplying PIs based on the product rule. The formulas of LRs calculation for trio and motherless cases are shown in Table S1. In this study, a PI greater than 10,000 was considered as proof of a parent–offspring relationship, where W, which represented the LR, corresponds to the probability of paternity being equal to or greater than 99.99%, assuming a priori probability of paternity of 0.5 [29]. When PI ranged from 0.0001 to 10,000 (including the aSTR mutation loci), other genetic markers were added until it was sufficient to make the decisions.

Due to the fact that there are limited data on Greek population genetics, the aSTR locus-specific mutation rates were collected based on the Caucasian population database provided by Ge J. et al. [31] and the PE value provided by Steffen C.R. et al. [28]. The overall loci-specific mutation rates with 95% confidence intervals (CI) were calculated at http://statpages.org/confint.html, accessed on 25 May 2009.

#### 2.4.2. Data Analysis for HLA Alleles

For the parentage investigations, incompatibilities in at least one HLA allele between the parents and offspring indicate exclusion. For inclusions, the statistical parameters paternity index (PI) and probability of paternity (W) were calculated using Essen–Möller values. The PI is the LR, which is calculated using the mathematical formula LR = 1/*p* (where *p* is the frequency of the HLA haplotype), assuming a prior probability of 0.5. The posterior probability of paternity, denoted as W by Essen–Möller, is then calculated using the W = LR/(LR + 1) formula [32]. The LR calculations were based on the HLA allele frequencies as estimated using the Caucasian population from the Allele Frequency Net Database (http://www.allelefrequencies.net/default.asp, accessed 2020). For the HLA genes, if no frequency data were available for the 3rd field, the frequency of the 2nd field was applied.

## 3. Results

### 3.1. aSTR Typing Results

Eighteen aSTR mutational events were observed and were distributed among the different loci as follows: five mutations on the SE33 locus, three mutations on the vWA locus; three mutations on the D12S391 locus; two mutations on the D8S1179 locus; and one mutation each on the D10S1248, D3S1358, FGA, D2S1338, and D19S433 loci. These mutations occurred in an equal number (18/428, 4.21%) of distinct parent–offspring allelic transfers, with two cases involving motherless scenarios. Notably, no mutations were detected in the other seven aSTR loci (refer to Table S2). The apparent mutation events were counted under approximately 708 meiotic transfers, resulting in 11 328 allele transfers in the parent–child duos, all of which were in the male germ line, which provided either a gain or loss of a single-step repeat unit. The ratio of repeat gains and losses was relatively balanced (5:9), while four mutations could not be assigned. The average paternal mutation rate estimated across all loci was 0.0016 (95% CI 0.0009–0.0025) per locus per gamete per generation, which is mostly in agreement with previous studies [33,34]. The genotype details of paternity inconsistencies that resulted from mutations in 16 autosomal microsatellite loci studied are described in Table 1 (unpublished data).

In 15 out of the 18 cases, the aSTR analysis of the results showed the alleged father (AF) could be determined as being the biological father with a probability value that ranged from 47,478 to Log_10_PI 3.98 × 10^9^. Only in three cases, the probability (W) was lower than 0.9999. Particularly, in the 11th case, the total PI value (8473) represented a likelihood that the genetic data supported the hypothesis of parentage over the hypothesis of coincidental paternal obligate allele(s) (POAs) sharing (when the PI value is between 1000 and 10,000, the verbal equivalent is “strong support”). Additionally, in the 8th and 14th cases, which were motherless, the PI values 697 and 7035, respectively, were weak data to support the hypothesis of parentage and more genetic markers are required for confident paternity results. Accordingly, in all cases, the indicators combined PE, which depends on deducing, in each case, the POAs from the child (in a duo) or the child plus its mother (in a trio), ranged from 0.99999978 to 0.99999999, and the RMNE, which ranged from Log_10_RMNE 1.82 × 10^−14^ to 1.29 × 10^−7^ (reliable equations to determine the power of a genetic test to exclude a pair of individuals as parents), argued in favor of relatedness (Table 2).

The 18 actual included cases were restudied after omitting maternal genotypes (i.e., only types of the father–child pairs were considered) to assess the probability of false exclusion occurrence upon simulating them into motherless cases. In two simulated cases (cases 15 and 16), no mismatches were detected, increasing the probability of relatedness (log_10_PI: 1.64 × 10^5^ and 5.25 × 10^6^, respectively), and therefore, fatherhood was not ruled out. Additionally, six included simulated duos failed to meet the criteria for concluding and reporting paternity inclusion, with PIs below the threshold of 10,000 (Table 2).

### 3.2. HLA Typing Results

#### 3.2.1. HLA Sequencing Metrics

**Figure 2:**
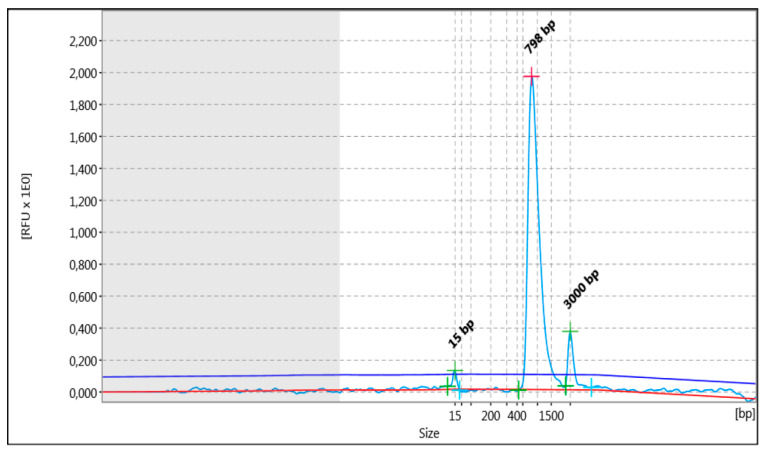
The analysis pertained to the distribution of fragments (light blue line) that underwent shearing, as observed in the entirety of the library being studied. The uppermost section of the graph depicts the fragment size evaluated (bp). In addition to the peaks, the detected baseline (in red) and the threshold for peak detection above the baseline (in blue) are displayed in the electropherogram.

**Figure 3:**
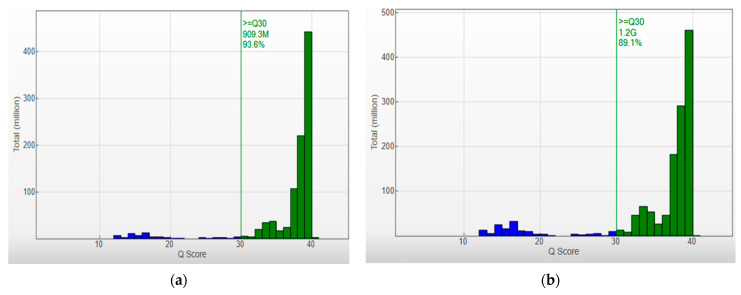
Q-score Distribution Analysis: Capturing Q-score distribution-graphical representations unveil the quantity of reads categorized by their respective quality scores. Q-scores below 30 are depicted in blue, and those between 30 and 40 are represented in green. Quality Metrics for HLA Sequencing: (**a**) Cluster density (K/mm^2^) = 715 ± 6, and clusters passing filter (%) = 94.11 ± 2.08. (**b**) Cluster density (K/mm^2^) = 990 ± 26, and clusters passing filter (%) = 87.73 ± 4.13. The median quality ≥ Q30 scores were 93.6% and 89.1%, respectively.

**Figure 4:**
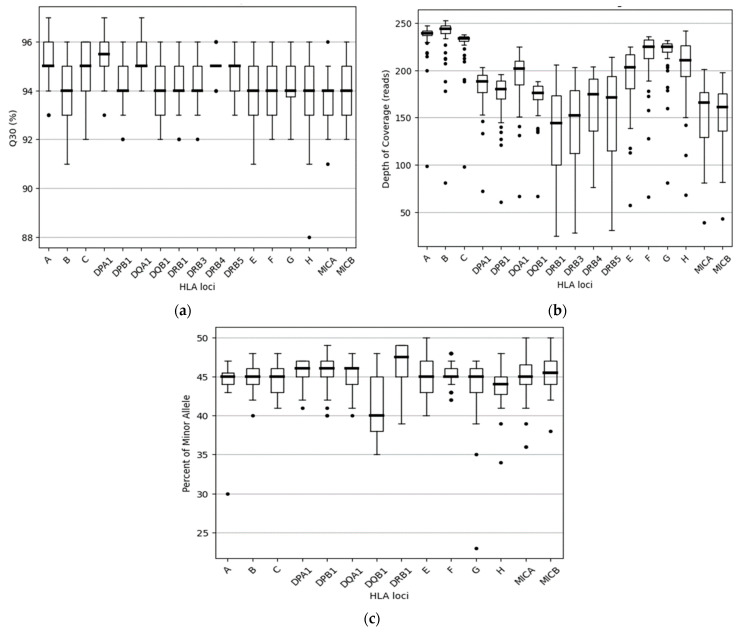
Distribution of Q30, usable reads, and percent of minor allele among the 17 HLA loci: (**a**) The percentage of base calls with a quality score above Q30 for 17 HLA genes, as observed using boxplots. Each box indicates the median and the first and third quartiles of the data, except for *-DRB4*, where similar values were observed. (**b**) The distribution of usable reads among the 17 HLA loci sequenced. Whiskers correspond to the interquartile range, and outliers are plotted as dots. (**c**) The distribution of allele balance for all heterozygous 14 HLA loci sequenced. Notably, the genes *HLA-DRB3/4/5* were excluded from this analysis due to the limited number of heterozygotes available for these genes. Outliers are also identified and plotted as dots.

In the above cases, in order to increase the strength of the genetic evidence, further analysis of samples using HLA typing on the Illumina MiSeq system was performed. In particular, 52 individuals were sequenced in two different runs (2 × 150 bp), and a total of 2109.3 MB of data was obtained. Τhe final pooled library concentrations were 15.2 ng/μL and 12.6 ng/μL (Figure 2). The run quality control (QC) metrics revealed a median cluster density of 715 K/mm^2^ (first run) and 990 K/mm^2^ (second run), with 94.1% and 87.7% passing filters (Figure 3a,b). The median quality ≥ Q30 scores were 93.6% and 89.1%, respectively; Figure 4a). The average depth of coverage was 189× for all HLA loci (Figure 4b) and ranged from 99 (*HLA-DRB1*) to 248 (*HLA-B*). As indicated by the average minor allele percentages, the *HLA-DQB1* locus had the highest allele imbalance, while *HLA-A* and *F* had the lowest (Figure 4c).

#### 3.2.2. HLA Haplotypes

The comprehensive analysis revealed a total of 34 HLA inherited haplotypes on the *HLA-A*, *~B*, *~C*, *~DRB1/3/4/5*, *~DQA1*, *~DQB1*, *~DPA1*, *~DPB1*, *~F*, *~G*, *~H*, *~E*, *MICA*, and *MICB* loci. The HLA typing results for each sample are presented in Table S3. Furthermore, a total of 274 unique alleles at either the field 3 or 4 level were identified, out of which 270 were determined to possess sequences that conformed identically to those documented within the IMGT/HLA database (Table 3). The remaining four alleles were distinguished by the presence of single-nucleotide polymorphism (SNP) variants and considered as HLA novel alleles. Their full-length DNA sequences were deposited in GenBank (accession numbers: OQ357851, OQ885042, OQ885046, and OQ885045) and the IPDIMGT/HLA Database (submission numbers: HWS10065189, HWS10066175, HWS10066149, and HWS10066153) (Table 4). The names HLA-B*14:02:01:26, -B*35:580, -B*40:02:01:41, and -C*04:01:01:175 were officially assigned by the World Health Organization (WHO) Nomenclature Committee for Factors of the HLA System in May 2023. This follows the agreed policy that subject to the conditions stated in the most recent Nomenclature Report [35], names will be assigned to new sequences as they are identified. Lists of such new names will be published in the following WHO Nomenclature Report. One nucleotide substitution was observed in exon 5 of *HLA-B*, while the remaining mutations were located in the non-coding regions of the *HLA-B* (3′UTR) and *HLA-B* and *-C* (5′UTR) genes. The single nucleotide substitution in the novel allele HLA-B*35:580 resulted in an amino acid change from alanine (A) to valine (V) (non-synonymous mutations). All novel HLA alleles that appeared in parent–child pairs followed the heredity rule. To validate the putative novel alleles observed at the *HLA-B* and *-C* loci, HLA genotyping using a commercial NGSgo^®^-MX6-1 kit (GenDx, Utrecht, The Netherlands) was performed and the results confirmed all identified mutation sites.

Also, in our study, 22 ambiguities at an 8-digit level were detected. Their HLA sequences matched with several allele combinations that could not be excluded based on the sequence information obtained. HLA alleles that have identical nucleotide sequences across the exons that encode the peptide binding domains but may show polymorphisms outside it belong to the same HLA G group. The most ambiguities were observed in the *HLA-DPB* locus (11 *HLA-DPB1* genotype combinations, for a total of 26 individuals), owing to the length limitation of PCR amplification, followed by the *HLA-DQB* (4 genotype combinations, for a total 5 individuals), *-DRB1* (4), *-DRB3* (1 genotype combination, 2 individuals), *-A* (1), and *-C* (1) loci. No ambiguities were found on the *HLA-B*, *-DRB4/5*, *DQA*, *DPA*, *-F*, *-G*, *-H*, *-E*, *MICA*, and *MICB* loci (Table S3).

Our results show that in the total of 34 meioses from the 18 parentage assessments, two discrepancy events on the *HLA-E* loci and one on *HLA-DRB3* were observed (Table S3). Specifically, for the fourth case, no data were obtained for the *HLA-H* genetic locus in the child in question. The HLA typing was HLA-H*02:07:01:01 homozygous for the AF and HLA-H*02:05:01:03 homozygous for the biological mother. Subsequently, for the 12th case, the HLA typing was HLA-H*02:01:01:01 homozygous for the AF, HLA-H*02:03:02 and *02:05:01:01 heterozygous for the biological mother, and HLA-H*02:03:02 homozygous for the child in question. Additionally, for the 13th case, no data were obtained for the *HLA-DRB3* genetic locus in the child in question (HLA-DRB3*02:02:01 for the biological mother and AF). All discrepancies observed were linked to PCR challenges, where certain alleles had minimal amplification that fell below our analysis program’s threshold and led to a “no call” outcome. This threshold, which was an internal parameter in our analysis pipeline, distinguished heterozygous from potentially homozygous samples. The remaining 15 HLA loci were inherited following Mendelian inheritance.

#### 3.2.3. Distribution of the LR

**Figure 5:**
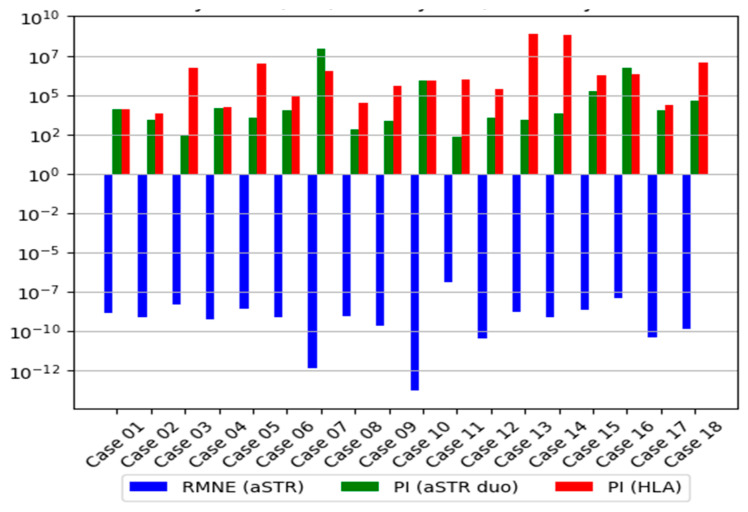
In this study, a PI of more than 10^4^ was considered proof of parent–offspring relationship. The median average Log_10_LRs of HLA genes were much higher than that of the aSTR loci in duo simulated cases, even when the HLA rare alleles on 3rd field or HLA novel alleles were not included in the analysis. Additionally, in all cases, the RMNE value illustrates the fact the genetic data supported the hypothesis of paternity.

The HLA genotyping revealed no mismatches between the child in question and the AF, giving a PI that ranged from 13,355 to Log_10_PI 8.06 × 10^8^ and a W that ranged from 0.99992513 to 0.99999999 regarding the six classical *HLA-A*, *-B*, *-C*, *-DRB1*, *-DQB1*, and *-DPB1* genes for the seventeen disputed cases (Table 2). For one case (case 2), HLA genotyping did not become sufficient to overcome the threshold of PI ≥ 10 000 (PI equal to 6 672) owing to the high frequency of HLA alleles transmitted by the AF (HLA-inherited haplotype ~A*29:02:01:01, ~B*35:01:01, ~C*04:01:01:06, ~DRB1*11:04:01G, ~DQB1*03:01:01:02, ~DPB1*04:01:01G). The genetic markers *HLA-DRB3/4/5*, *-DQA1*, *-DPA1*, *-F*, *-G*, *-H*, *-E*, *MICA*, and *MICB* were not included in the statistical analysis owing to limited haplotype frequency data in the Caucasian population. The HLA-inherited haplotypes transmitted by the AFs are shown in Table S3. Combining the LR values obtained from the two systems (16 aSTR markers and 6 linked HLA loci) and assuming a prior probability of 0.5, the resulting value of Log_10_CPI was more than 1.69 × 10^7^ and W was more than 0.99999994, which were sufficient for the verbal predicate “paternity practically proven” for all duo simulated cases (Figure 5).

## 4. Discussion

The aSTR analysis has demonstrated itself to be one of the most reliable and cost-effective molecular tools in forensic casework. However, aSTRs may undergo evident variations in the copy number through a process known as dynamic or mutable mutation, resulting in incompatibility between parents and offspring. If mutation events are overlooked, it can have a significant impact on the genetic evidence of consanguinity, potentially leading to a result supporting an incorrect conclusion [36]. If mutations are not considered, LR values might lead to false exclusion [37]. Additionally, in cases of inclusion, the mean frequency of mutation in the relevant population should be considered, as well as its range. This factor could potentially alter the data [31].

The aSTR data from the present study showed that fatherhood was not ruled out for 15 out of 16 complete triplet cases with isolated exclusions by mutations, and a sufficient paternity probability was achieved even after the mutation calculation, with an average probability of 0.99999979, Log_10_PI: 4.71 × 10^6^ (min. W: 0.99997894, PI 47,478; max. W 0.99999999, Log_10_PI: 3.98 × 10^9^). In one triplet case, the W for the biological father was only 0.99988199 (PI 8 473) due to the inheritance of very common aSTR alleles. In addition, when all the above triplet cases were further analyzed without investigation of the mother’s genetic profile from the probability calculation, this displayed a remarkable decrease in the simulated duo cases below 0.999 compared with that of the trio cases, and the difference became even more significant when the aSTR POAs were presented with high frequency in the population study [17]. Also, while in the two triplet cases, one discrepancy was shown between the AF and the child in question, this discrepancy was eliminated when the DNA profile of the mother was missing from the analysis, which was an event observed in other studies [38]. Our results led to the conclusion that when simulating the duo from trio families, the mother’s genetic profile can hide additional mismatches, providing enough certainty to include the putative father [39]. Additionally, we emphasize the necessity for greater caution when dealing with motherless cases, especially in cases where mutation events occur. Therefore, the present study evaluated the effect of the availability of both parents in cases with observed mutation events. The 16 forensic loci were sufficient to provide positive proof (strong evidence) of paternity, offering high discriminating power in only 15 out of 16 triplet cases, and more genetic information and accurate statistical analyses for achieving confident results are required. According to García-Aceves et al., the implementation of ≥ 20 aSTRs as the routine battery of markers for paternity testing labs allows for obtaining sound conclusions to solve the large majority of motherless cases [33]. In addition, as most DNA-typing applications have legal and ethical implications and there is a particular need for high reliability and high discrimination power in cases where fatherhood cannot be confirmed or ruled out with statistical certainty, the focus should be shifted to the application of alternative and often more discriminative and polymorphic markers [40,41,42].

HLA genotyping using MPS should be considered in cases where the complexity of the involved subjects requires a deeper analysis. Nonetheless, even if the HLA system is one of the most extensively studied regions, this level of polymorphism remains a challenge when it comes to type HLA genes. To date, with existing short-read technologies, HLA genotyping ambiguities generated due either to failure to interrogate all polymorphic positions or when two or more different allele combinations produce identical sequences (cis/trans ambiguities) remain an issue. As it is well known, the *HLA-DPB1* is the most susceptible to generating an ambiguous locus. In our study, almost all results, except *HLA-DPB1*, were obtained without any ambiguities at the third field level. Phasing of the large intron 2 of the *-DPB1* locus is of significant importance and could reduce the rate of ambiguities reported, leading to a more accurate description of HLA diversity [43]. Also, the high accuracy percentage obtained using MPS indicates adequate coverage that allows for correct HLA variant calls. In this study, acceptable quality results were observed in all HLA loci without allele dropout events, except on *HLA-E* and *HLA-DRB3* loci, where three discrepancy events due to low DNA concentration were observed. This is a known potential limitation of multiplex primers. However, in the case of failure or doubt in only one locus of a given sample, it is recommended to include the sample in another test battery or to perform additional testing [44].

HLA genotyping using MPS outside the core region (exon 2, 3, and 4 for HLA class I; exon 2 and 3 for HLA class II genes) gives more precise sequencing results and allows for the identification of rare and novel HLA alleles using the high-quality typing compared with conventional techniques [45,46,47]. As illustrated in the results section, four novel HLA alleles were identified for classical HLA class genes, leading to a more accurate HLA haplotype definition. It should be noticed that the median average Log_10_LRs of HLA genes were much higher than that of the aSTR loci in duo simulated cases, even when the rare alleles on the third field or novel alleles were excluded from the analysis, which led to reliable results similar to those achieved in complete triplet analysis using aSTRs.

In this report, the analysis primarily focused on classical HLA genes (*HLA-A*, *-B*, *-C*, *-DRB1/3/4/5*, *-DQA1*, *-DQB1*, *-DPA1*, *-DPB1*) and secondarily on non-classical HLA genes (*HLA-F*, *-G*, *-H*, *-E*), *MICA*, and *MICB*. Information about non-classical HLA genes allows for a more accurate description of HLA haplotype diversity but relatively few reference data exist on polymorphisms at the high-resolution level (third or fourth field) [48]. To accurately determine the relationship between individuals, it is crucial to have access to high-resolution HLA frequency data for different populations. While the number of newly discovered HLA alleles has increased significantly in recent years, the IMGT/HLA reference database does not have complete genomic sequences for all HLA class II alleles, which leads to the generation of ambiguous results. Ehrenberg et al. primarily attributed these challenges to factors such as inadequate reference data, the emergence of new SNPs in less-studied populations, and distinguishing SNPs within the untranslated region (UTR) [49]. In the present study, the LR calculations were based upon HLA allele frequencies, as estimated from the Caucasian population from the Allele Frequency Net Database, as our population has not been adequately standardized at the third field level at present. Our results demonstrate that even when no frequency data were available in the third field in most of the cases, the median average Log_10_LRs (min. W 0.99985014, PI 6672; max. W 0.99999999, Log_10_PI 8.06 × 10^8^) of the HLA genes managed to reach definitive conclusions and improve the power of discrimination for kinship determination in comparison with the aSTR analysis in seventeen out of the eighteen parentage cases. Finally, combining the LR values obtained from the two systems (aSTR markers and six linked HLA loci), the arrived value of Log_10_PI was 1.69 × 10^7^, which was sufficient for the verbal predicate “paternity practically proven” for the eighteenth simulated duo case.

The rapid advancement of MPS technology has significantly enhanced its clinical application in precision medicine, revolutionizing the research of genetic factors in structural disorders [50]. Especially, in immunogenetics, MPS has emerged as a powerful and versatile tool, offering efficient and cost-effective DNA/RNA sequencing while surpassing the limitations of traditional methods [21,51]. In forensic applications, HLA typing is highly recommended by parentage profiling laboratories for cases involving individuals without a known mother or father, especially when an aSTR mutation event is recorded. The utilization of MPS for HLA genotyping can augment the LR in identifying true parental relationships within a pedigree, while concurrently minimizing the risk of erroneous attributions. Furthermore, the unique genomic characteristics of HLA genes located in proximity on the same chromosome and inherited as haplotypes in a Mendelian manner render them exceptionally conducive to parentage testing. The intricate patterns of inheritance associated with HLA genes make them particularly pertinent for discerning relationships between individuals originating from the same lineage or for undertaking indirect kinship analyses [42,52].

To fully harness the potential of the HLA system in disputed paternity/maternity assessments, certain considerations need to be addressed. These include evaluating the relevance of the provided information, the ability to analyze the complete HLA gene across all genetic loci, and the availability of high-resolution HLA frequency data across diverse populations. While acknowledging the constraints imposed by current limitations in population haplotypic databases, it is essential to underscore the substantial contributions made by MPS in enhancing the specificity and discriminatory power of HLA gene analysis, thereby significantly fortifying the robustness of paternity testing. The selection of MPS technology for HLA gene analysis is underpinned by its capacity to provide a more exhaustive and intricate characterization of genetic variations. By adequately addressing these issues, we can facilitate a comprehensive reintegration of the HLA system, thereby reinforcing its application in disputed paternity/maternity assessments.

However, the incorporation of HLA alleles into the field of forensic identification should not be perceived as a substitution but rather as a supplementary tool to complement the well-established and extensively utilized markers already in place [53]. The integration of MPS for the analysis of HLA genes in paternity testing offers a complementary approach alongside conventional forensic kits, which primarily focus on STRs and SNPs. While traditional kits have demonstrated efficacy in forensic applications, the integration of MPS provides an additional layer of depth to the genetic analysis. MPS allows for a more comprehensive sequencing of HLA genes, enabling the identification of a broader spectrum of genetic variations, including indels, copy number variations, and complex structural variations. This simultaneous use of both methodologies harnesses the strengths of each approach. Forensic kits, with their known sequences of STR alleles, enhance the statistical values of results. On the other hand, MPS contributes to a finer understanding of genetic relationships, offering increased resolution and discrimination. The parallel application of these methodologies ensures a more robust and versatile paternity-testing framework that combines the strengths of traditional forensic approaches with the detailed insights provided by MPS technology.

## 5. Conclusions

In conclusion, despite their limitations, aSTRs remain the most reliable and widespread markers used in forensic identification. However, in instances where conventional methods prove insufficient in yielding satisfactory results, there arises a need for alternative or supplementary markers to facilitate the analysis. The inclusion of HLA genotyping using MPS technology presents a promising avenue to overcome these limitations. By harnessing the capabilities of MPS, HLA polymorphism analysis holds the potential to enhance the evaluation of relatedness in cases involving genetic deficiencies. These insights highlight the necessity for further exploration and optimization of HLA genotyping methods, ultimately leading to improved accuracy and reliability in paternity determination and relatedness assessment. Concurrently, the reintroduction of HLA alleles into the sphere of forensic identification must be perceived as an auxiliary tool to complement preexisting and widely accepted markers. This symbiotic union of HLA genes and established forensic markers holds the promise of engendering an exceptionally potent methodology for future applications within the domain of forensic analysis.

## Figures and Tables

**Table 1:** Eighteen aSTR mutations were observed in equal different parent/child allele transfers. Paternity not excluded.

	aSTRs Loci	Alleged Father Genotype	Mother Genotype	Child Genotype	POA	Putative Mutation	Gain/Loss	Sex/Age (Years)	Single/Double Step
Case 01	SE33	18, 20	20, 34	19, 34	18 or 20	18→19 οr 20→19	Gain or loss	Male/45	Single
Case 02	SE33	15, 34	12, 16	16, 16	15	15→16	Gain	Male/29	Single
Case 03	SE33	17, 25.2	19, 28.2	19, 24.2	25.2	25.2→24.2	Loss	Male/39	Single
Case 04	SE33	28.2, 33.2	22.2, 28.2	28.2, 32.2	33.2	33.2→32.2	Loss	Male/41	Single
Case 05	SE33	20, 20	16, 32.2	19, 32.2	20	20→19	Loss	Male/39	Single
Case 06	vWA	17, 19	16, 18	18, 20	19	19→20	Gain	Male/41	Single
Case 07	vWA	16, 19	16, 17	17, 18	19	19→18	Loss	Male/36	Single
Case 08	vWA	17, 20	NA	18, 19	Uncertain	Uncertain	Gain or loss	Male/33	Single
Case 09	D12S391	16, 21	22, 22	20, 22	21	21→20	Loss	Male/31	Single
Case 10	D12S391	21, 24	20, 20	20, 20	21	21→20	Loss	Male/35	Single
Case 11	D12S391	22, 24	19.3, 22	19.3, 23	22 or 24	22→23 οr 24→23	Gain or loss	Male/28	Single
Case 12	D8S1179	13, 13	12, 12	12, 14	13	13→14	Gain	Male/49	Single
Case 13	D8S1179	13, 15	8, 13	8, 16	15	15→16	Gain	Male/19	Single
Case 14	D10S1248	14, 17	NA	15, 16	Uncertain	Uncertain	Gain or loss	Male/45	Single
Case 15	D3S1358	15, 18	18, 18	18, 19	18	18→19	Gain	Male/41	Single
Case 16	FGA	19, 23	20, 23	22, 23	23	23→22	Loss	Male/35	Single
Case 17	D2S1338	16, 24	24, 25	23, 25	24	24→23	Loss	Male/40	Single
Case 18	D19S433	15, 15	13, 14.2	13, 13	15	15→13	Loss	Male/27	Double

NA, not available; POA, paternal obligate allele.

**Table 2:** Random man not excluded (RMNE), power of exclusion (PE), combined paternity index (PI), and probability of paternity (W) for autosomal STRs (aSTRs) and human leukocyte antigen (HLA) alleles.

	aSTR Trio				aSTR Duo *		HLA		aSTR Duo and HLA	
	RMNE	PE	PI	W	PI	W	PI	W	PI	W
Case 01	1.39 × 10^−9^	0.99999999	6.66 × 10^5^	0.99999849	11,732	0.99991477	13,355	0.99992513	1.57 × 10^8^	0.99999999
Case 02	7.82 × 10^−10^	0.99999999	1.22 × 10^5^	0.99999179	2540	0.99960645	6672	0.99985014	1.69 × 10^7^	0.99999994
Case 03	4.90 × 10^−9^	0.99999999	5.91 × 10^5^	0.99999830	260	0.99616858	5.04 × 10^6^	0.99999980	1.31 × 10^9^	0.99999999
Case 04	5.54 × 10^−10^	0.99999999	3.78 × 10^6^	0.99999973	13,925	0.99992819	16,193	0.99993825	2.25 × 10^8^	0.99999999
Case 05	2.85 × 10^−9^	0.99999999	1.72 × 10^5^	0.99999418	3769	0.99973475	9.63 × 10^6^	0.99999989	3.63 × 10^10^	0.99999999
Case 06	8.17 × 10^−10^	0.99999999	2.43 × 10^7^	0.99999995	10,798	0.99990740	81,867	0.99998778	8.83 × 10^8^	0.99999999
Case 07	4.81 × 10^−13^	0.99999999	3.98 × 10^9^	0.99999999	9.25 × 10^7^	0.99999999	3.48 × 10^6^	0.99999971	3.21 × 10^14^	0.99999999
Case 08	9.68 × 10^−10^	0.99999999	NA	NA	697	0.99856734	30,769	0.99996750	2.14 × 10^7^	0.99999995
Case 09	2.14 × 10^−10^	0.99999999	1.29 × 10^5^	0.99999224	2402	0.99958385	4.07 × 10^5^	0.99999754	9.77 × 10^8^	0.99999999
Case 10	1.82 × 10^−14^	0.99999999	4.79 × 10^7^	0.99999998	8.35 × 10^5^	0.99999880	8.72 × 10^5^	0.99999885	7.28 × 10^11^	0.99999999
Case 11	1.29 × 10^−7^	0.99999978	8473	0.99988199	210	0.99526066	1.03 × 10^6^	0.99999903	2.16 × 10^8^	0.99999999
Case 12	3.64 × 10^−11^	0.99999999	5.44 × 10^6^	0.99999982	3631	0.99972467	2.53 × 10^5^	0.99999605	9.19 × 10^8^	0.99999999
Case 13	1.57 × 10^−9^	0.99999999	4.88 × 10^5^	0.99999795	2864	0.99965096	8.06 × 10^8^	0.99999999	2.31 × 10^12^	0.99999999
Case 14	7.38 × 10^−10^	0.99999999	NA	NA	7035	0.99985787	6.34 × 10^8^	0.99999999	4.46 × 10^12^	0.99999999
Case 15	2.44 × 10^−9^	0.99999999	51,493	0.99998058	1.64 × 10^5^	0.99999391	1.72 × 10^6^	0.99999941	2.83 × 10^11^	0.99999999
Case 16	1.23 × 10^−8^	0.99999998	85,436	0.99998829	5.25 × 10^6^	0.99999981	2.19 × 10^6^	0.99999954	1.15 × 10^13^	0.99999999
Case 17	4.01 × 10^−11^	0.99999999	47,478	0.99997894	11,035	0.99990939	21,851	0.99995424	2.41 × 10^8^	0.99999999
Case 18	1.45 × 10^−10^	0.99999999	6.02 × 10^7^	0.99999998	40,886	0.99997554	1.23 × 10^7^	0.99999999	5.04 × 10^12^	0.99999999

* Duo simulated cases from the previous trio family.

**Table 3:** HLA alleles identified in this study.

HLA Locus	A	B	C	E	F	G	H			
Alleles	*01:01:01:01* *02:01:01* *02:01:01:01* *02:01:01:05* *02:02:01:01* *02:11:01:01* *03:01:01:01* *03:02:01:01* *11:01:01* *11:01:01:01* *23:01:01:01* *24:02:01:01* *24:02:01:04* *26:01:01:01* *29:02:01:01* *30:01:01:01* *30:04:01:01* *31:01:02:01* *32:01:01:01* *33:01:01:01* *68:01:01:02* *68:01:02:02* *69:01:01:01*	*07:02:01* *07:05:01* *13:02:01:01* *14:02:01:26* * 15:18:01:02 *18:01:01* *18:05:01:02* *27:05:02:01* *35:01:01* *35:03:01* *35:03:01:01* *35:03:01:03* *35:08:01* *35:08:01:01* *35:580* * 37:01:01 *38:01:01* *39:01:01:05* *40:01:02* *40:02:01:41* * 40:06:01:13 *41:01:01:01* *41:02:01:01* *44:02:01* *44:02:01:01* *44:05:01:01* *49:01:01:01* *49:01:01:04* *51:01:01* *51:01:01:01* *51:01:01:10* *51:01:01:11* *51:01:01:33* *51:07:01* *51:08:01:01* *52:01:01:01* *52:01:01:02* *52:01:01:18* *55:01:01:01* *56:01:01* *58:01:01:03*	*01:02:01* *01:02:01:01* *02:02:02:01* *03:02:02* *03:02:02:05* *03:03:01:01* *03:04:01:01* *04:01:01:06* *04:01:01:14* *04:01:01:175* * 04:01:01:75 *04:01:01:79* *05:01:01* *05:01:01:02* *06:02:01:01* *07:01:01* *07:01:01:16* *07:02:01* *07:02:01:01* *07:02:01:03* *07:04:01:03* *08:02:01:01* *12:02:02* *12:02:02:01* *12:03:01:01* *14:02:01:01* *15:02:01* *15:02:01:01* *15:05:02* *15:09:01:01* *16:02:01:01* *16:04:01:01* *17:01:01:05*	*01:01:01* *01:01:01:06* *01:01:01:10* *01:03:01:01* *01:03:02* *01:03:02:01* *01:03:03* *01:03:05:01* *01:06:01:01*	*01:01:01* *01:01:01:08* *01:01:01:09* *01:01:01:18* *01:01:02* *01:01:02:08* *01:01:02:09* *01:01:02:10* *01:03:01* *01:03:01:03* *01:04:01:02*	*01:01:01* *01:01:01:01* *01:01:01:04* *01:01:01:05* *01:01:02* *01:01:02:01* *01:01:03:03* *01:01:08* *01:01:12* *01:01:13* *01:01:14* *01:01:22:01* *01:03:01* *01:03:01:02* *01:04:01:01* *01:04:04* *01:05N* *01:06:01:01*	*01:01:01* *01:01:01:01* *01:01:02:01* *01:03:01:01* *02:01:01:01* *02:02:01:01* *02:03:01* *02:03:02* *02:04:01* *02:05:01:01* *02:05:01:03* *02:07* *02:07:01:01* *02:09* *02:12* *02:23*			
**HLA Locus**	**DPA1**	**DPB1**	**DQA1**	**DQB1**	**DRB1**	**DRB3**	**DRB4**	**DRB5**	**MICA**	**MICB**
Alleles	*01:03:01* *01:03:01:60* *02:01:01* *02:01:01:02* *02:01:08* *02:07:01*	*01:01:01G* *02:01:02* *02:01:02G* *03:01:01G* *04:01:01* *04:01:01G* *04:02:01* *04:02:01G* *06:01:01G* *10:01:01G* *104:01:01* *13:01:01* *13:01:01G* *14:01:01G* *23:01:01*	*01:01:01* *01:01:02:01* *01:02:01* *01:02:02* *01:03:01* *01:03:01:01* *01:04:01* *01:04:02* *01:05:01* *02:01:01* *02:02:02* *03:01:01* *03:03:01* *05:01:01* *05:01:01:03* *05:05:01* *06:01:01* *06:01:01:03*	*02:01:01* *02:02:01* *02:02:01:02* *03:01:01* *03:01:01:02* *03:01:01G* *03:02:01* *03:03:02* *03:292* *05:01:01* *05:01:01G* *05:02:01* *05:03:01* *05:03:01G* *06:01:01* *06:02:01* *06:03:01* *06:03:01G* *06:04:01* *06:04:01G*	*01:01:01* *01:02:01:03* *03:01:01* *04:01:01* *04:02:01* *04:05:01* *04:07:01* *07:01:01* *07:07:01:01* *08:01:01* *08:03:02* *08:10* *10:01:01* *11:01:01* *11:03:01* *11:04:01* *11:04:01G* *12:01:01* *13:01:01* *13:01:01:07* *13:02:01* *14:01:01G* *14:04:01* *14:54:01* *15:01:01* *15:02:01* *15:02:02* *16:01:01* *16:02:01G*	*01:01:02* *01:01:02G* *02:02:01* *02:02:01G* *03:01:01*	*01:01:01* *01:03:01* *01:03:01N*	*01:01:01* *01:02:01* *01:10N* *02:02:01*	*002:01* *004:01* *006* *007:01* *008:01* *008:02* *008:04* *009:01* *011:01* *012:01* *012:03* *016:01* *018:01* *019:01* *027:01* *049:01* *19:01*	*002:01* *004:01* *005:01* *005:02* *005:03* *008:01* *014:01*

* Novel allele; uppercase “G”, ambiguous allele; uppercase “N”, null allele.

**Table 4:** HLA novel alleles detected in 52 individuals.

HLA Novel Allele	Most Homologous Allele	gDNA Position	Exon	Codon Change	NC	SNP	aa Change	Type of Mutation	GenBank Accession Number	IPD-IMGT/HLA Database Accession Number
B*14:02:01:26	B*14:02:01:01	−260			5′UTR	G to T	No	Silent	OQ357851	HWS10065189
B*35:580	B*35:01:01:02	2023	5	304 (GCT >> GTT)		C to T	Yes Ala (A) to Val (V)	Non-synonymous	OQ885042	HWS10066175
B*40:02:01:41	B*40:02:01:01	3010:1 3020 3021			3′UTR	C to Deletion (−) C to T C to T	No	Silent	OQ885046	HWS10066149
*C* *04 *:01:01:175*	*C* *04 *:01:01:01*	−249			5′UTR	C to T	No	Silent	OQ885045	HWS10066153

aa, amino acid; SNP, single-nucleotide polymorphism; NC, non-coding region.

## Data Availability

The data that support the findings of this study are available on request from the corresponding author. The data are not publicly available due to privacy or ethical restrictions. Data on *HLA-B*14:02:01:26*, *-B*35:580*, *-B*40:02:01:41*, and *-C*04:01:01:175 novel alleles are* freely available in the GenBank Database (https://www.ncbi.nlm.nih.gov/genbank/) (accession numbers: OQ357851, OQ885042, OQ885046, and OQ885045, respectively) and the IPD-IMGT/HLA Database (https://www.ebi.ac.uk/ipd/imgt/hla/) (submission numbers: HWS10065189, HWS10066175, HWS10066149, and HWS10066153, respectively).

## Supplementary Materials

The following supporting information can be downloaded at: https://www.mdpi.com/article/10.3390/genes15020150/s1, Table S1: The formulas of likelihood ratio (LR) calculation for trio and motherless cases; Table S2: aSTR typing results; Table S3: Classical and non-classical HLA typing results.
